# Supplementary material for: The differential effect of SARS-CoV-2 NSP1 on mRNA translation and stability reveals new insights linking ribosome recruitment, codon usage, and virus evolution
Source: Nucleic Acids Res. 2025 Apr 7;53(6):gkaf261. doi: 10.1093/nar/gkaf261 (PMC11975289; doi:10.1093/nar/gkaf261)
Supplement: gkaf261_Supplemental_File [file gkaf261_supplemental_file.pdf]

The differential effect of SARS-COV-2 NSP1 on mRNA translation and stability reveals new insights linking ribosome recruitment, codon usage and virus evolution

Supplemental information

Figure S1
Figure S2
Figure S3
Figure S4
Figure S5
Figure S6
Figure S7
Table S1
Table S2
Table S3

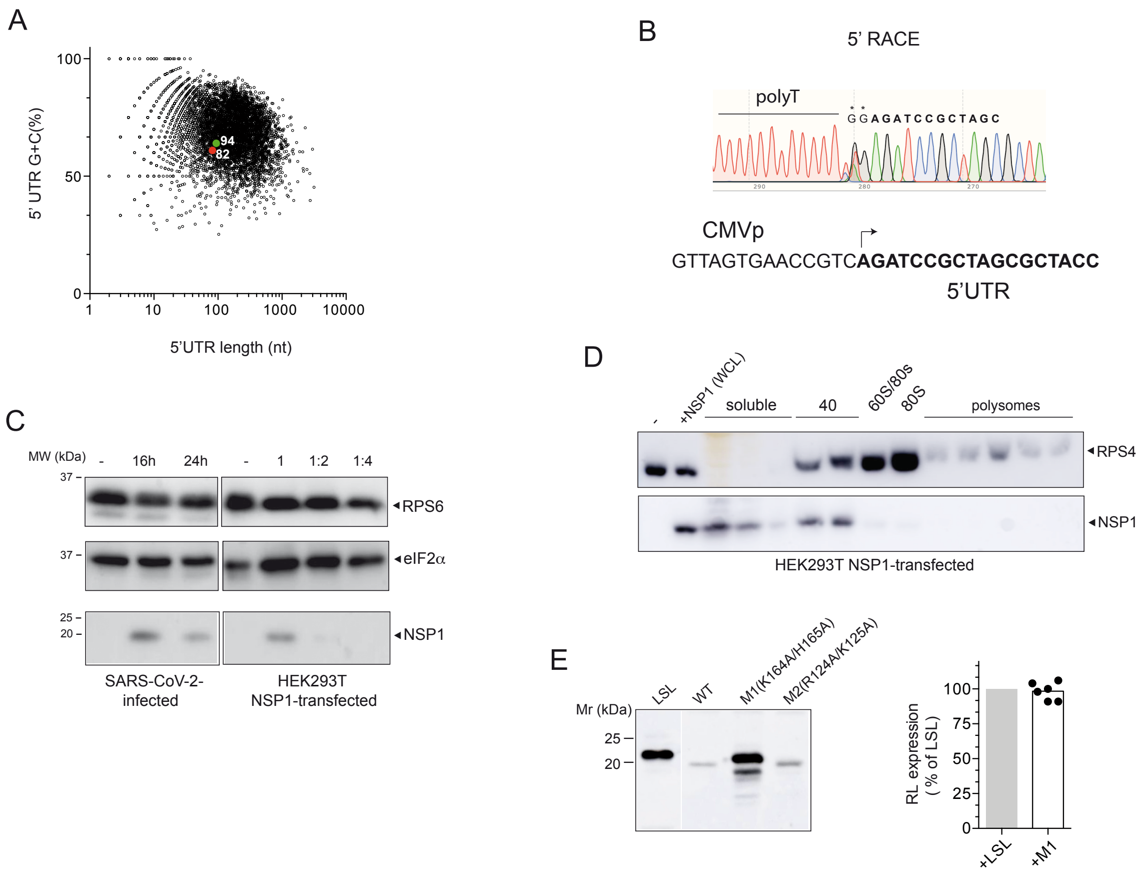

Figure S1. (A) Plot of length and G+C composition of human 5' UTRs previously identified by Nanocage technology (#REF) that were used in this study. The positions of parental 94- and 82-5' UTR of reporter mRNAs are shown. (B) Mapping the 5' end of 82-RL mRNA by 5' RACE. The sequencing chromatogram is shown. Asterisks denote the extra GG that results from known template-independent addition of two Cs by reverse transcriptase during cDNA synthesis (#REF Zajac et al). The mapped transcription start site (arrow) matched with that described for parental pEGFP-N1(Clontech). (C) Comparative analysis of NSP1 levels accumulated in HEK293T cells transfected with pNSP1-WT plasmid and VeroE6 infected with SARS-CoV-2. The 1/2 and 1/4 dilutions of HEK293T extracts are indicated. Blots were probed with the indicated antibodies. (D) Association of NSP1 to 40S subunits in transfected HEK293T cells. Fractions from polysome analysis were probed with anti-NSP1 and anti-RPS4 antibodies. The positions of the resulting identified peaks are shown. (E) NSP1-M1 is a *null* mutant showing not detectable effect on RL expression. The effect of NSP1-M1 on RL expression was compared to that of an unrelated protein (LSL, mushroom lectin protein, right panel). A western-blot showing the expression of the corresponding genes in HEK293T cells is also shown (left panel).

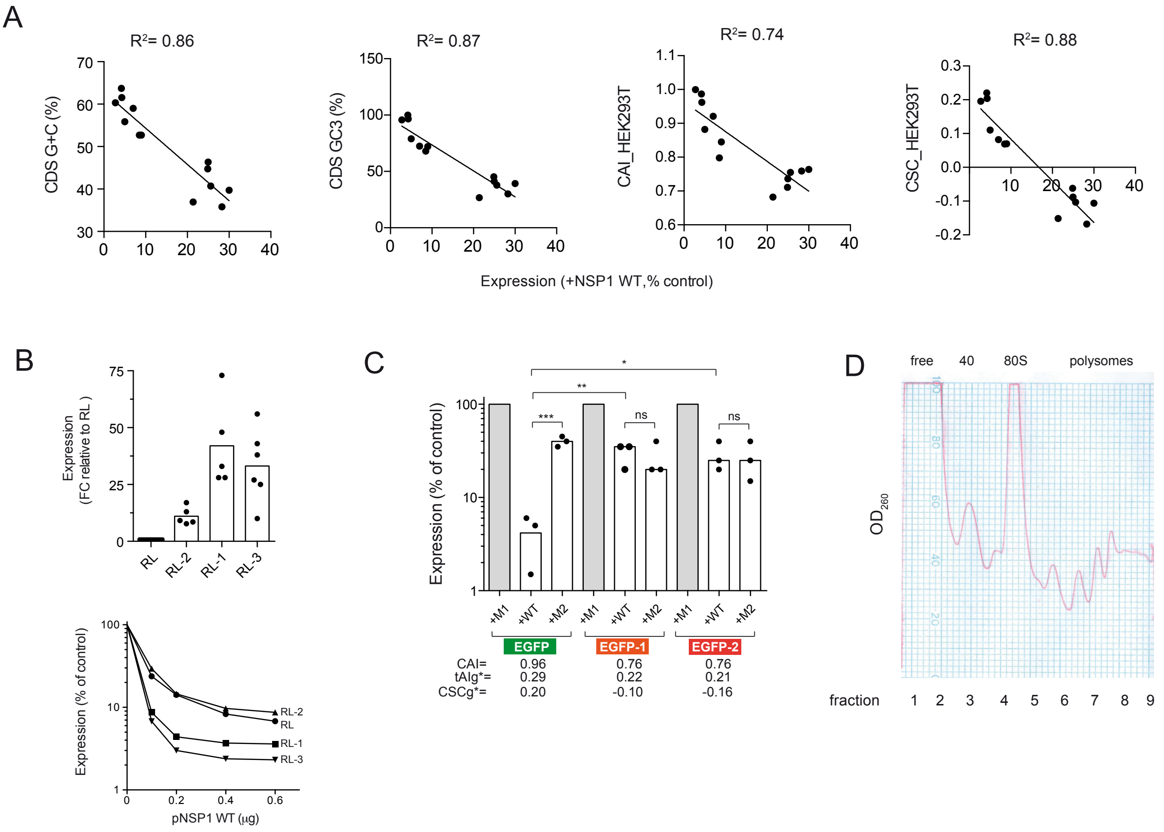

Figure S2. (A) Correlation between sensitivity to NSP1-WT and reporter's CDS features including G+C (%), GC3 (%), CAI and CSCg estimated for HEK293T cells. The resulting Pearson R^2 is shown. (B) Expression of RL variants (RL-1 to RL-3) with different codon usage bias. Data are expressed as fold change (FC) relative to RL expression (left panel). Effect of increasing amount of pNSP1 WT plasmid on expression of RL variants (right panel). (C) Effect of EGFP CDS recoding on sensitivity to NSP1 WT and M2. The CAI, tAIg and CSCg values for parental (EGFP, green) and EGFP-1 and EGFP-2 (orange to red) are indicated. Significance was scored as described in Materials and Methods. (D) Polysome profile of experiment showed in figure 2C.

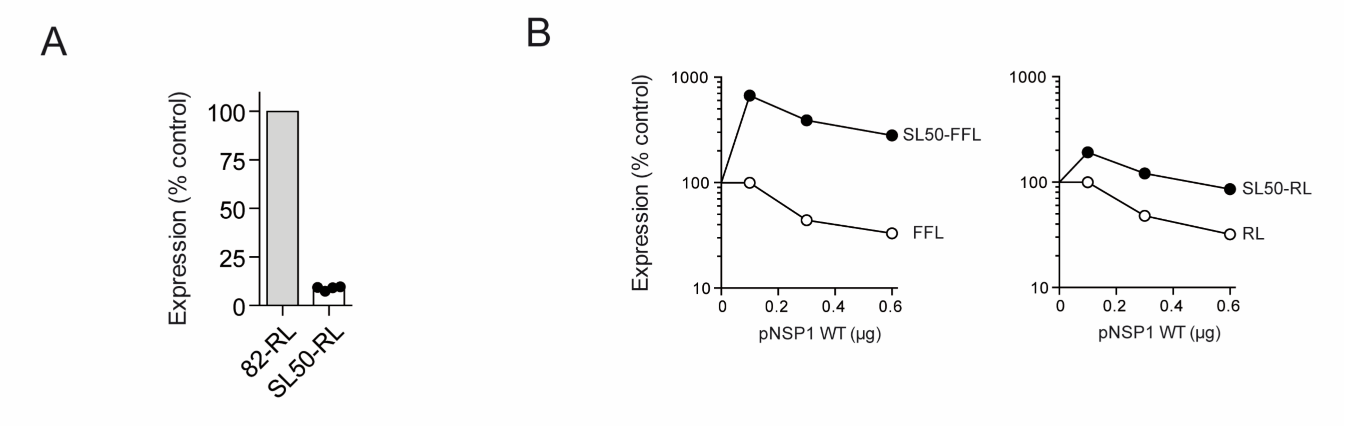

Figure S3. (A) Effect of upstream SL50 on RL expression. Data are expressed relative to parental 82-RL (100%). (B) Comparative analysis of the effect of increasing amounts of pNSP1 WT plasmid on expression of SL50-FFL and SL50-RL relative to pNSP1 M1(100%).

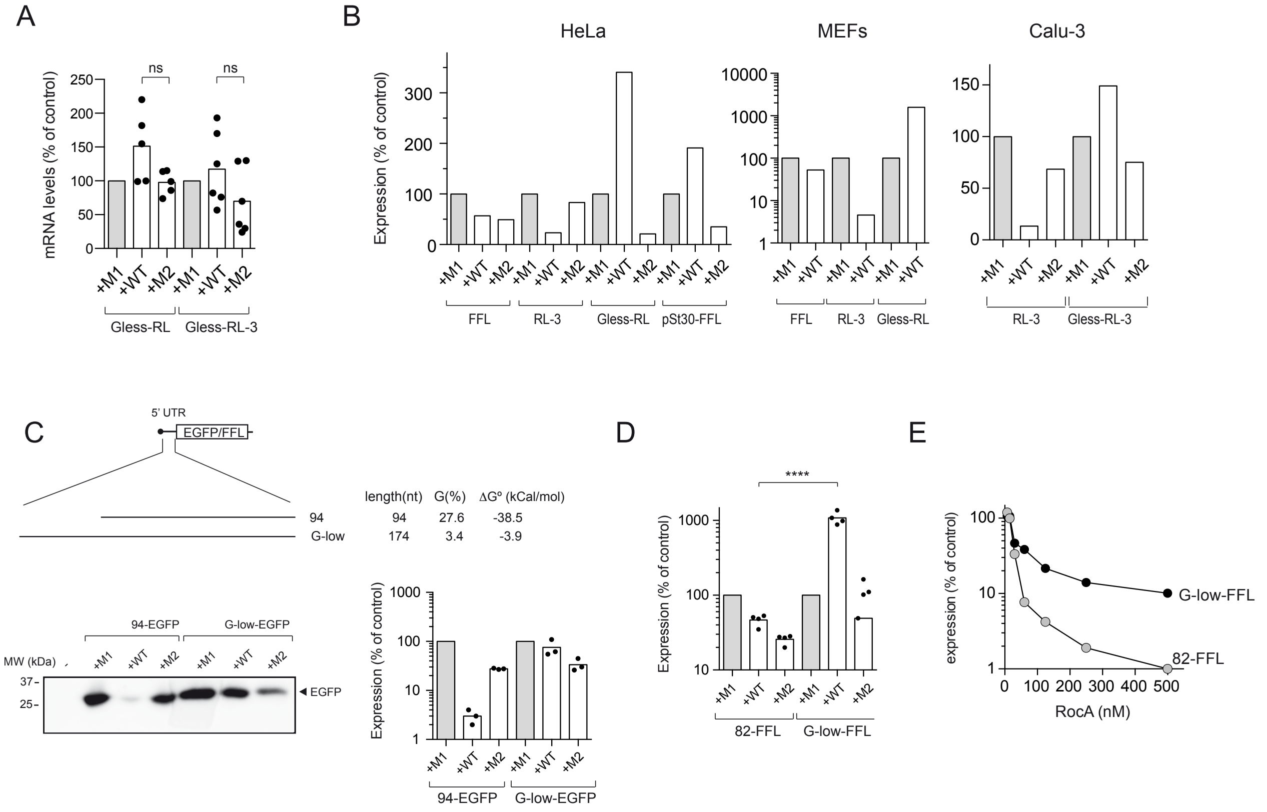

Figure S4. (A) Relative mRNA levels of G-less-RL and G-less-RL-3 accumulated in cells co-expressing NSP1 WT or M2. Data were expressed and significance was scored as described in Figure 1. (B) Effect of NSP1 WT and M2 on expression of the indicated reporters in HeLa, MEF and Calu-3 cells. Data are the mean from two (HeLa and MEFs) or three (Calu-3) independent experiments. (C) Effect of G-low 5' UTR on the sensitivity of EGFP and FFL to NSP1 WT and M2. Features of 94 and G-less 5' UTRs including length, number of Gs and stability (ΔGº in kcal/mol) are indicated (upper panel). A representative western blot of EGFP accumulated in cells cotransfected with the indicated constructs and the resulting quantification from three independent experiments are shown (bottom panels). (D) Influence of G-low 5' UTR on the sensitivity of FFL to NSP1 WT and M2. Data are expressed and significance was scored as described in previous figures. (E) Comparative analysis of the sensitivity of 82-FFL and G-low-FFL to RocA. Inhibitor addition and measurements were taken as described above.

A

	G+C(%)	GC3(%)	tAIg	CSCg	CAI
SARS-CoV-1	41.0	35.0	0.21	-0.10	0.77
SARS-CoV-2	37.9	28.1	0.21	-0.14	0.76
MERS	41.4	35.8	0.21	-0.10	0.77
hCoV-OC43	36.8	27.8	0.21	-0.14	0.77
hCoV-HKU1	32.3	18.6	0.20	-0.19	0.77
hCoV-229E	38.2	30.3	0.22	-0.13	0.77
hCoV-NL63	34.4	21.4	0.21	-0.17	0.76
N_SARS-CoV-2	46.3	38.0	0.21	-0.08	0.73
ACTB	60.5	84.5	0.26	0.12	0.89

B

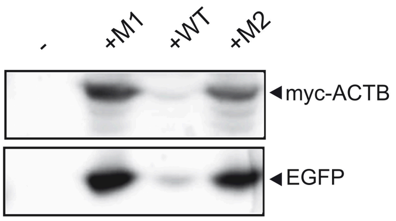

C

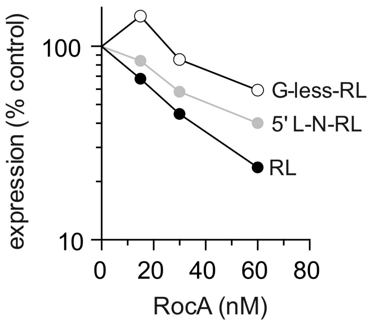

Figure S5. (A) Nucleotide composition and codon usage metrics of coding sequences of representative human coronavirus and ACTB mRNA. (B) Sensitivity of human ACTB to NSP1 and its mutants. A representative western blot of myc-ACTB and EGFP is shown. (C) Comparative analysis of the differential sensitivity of RL, G-less-RL and 5' L-RL expression to RocA.

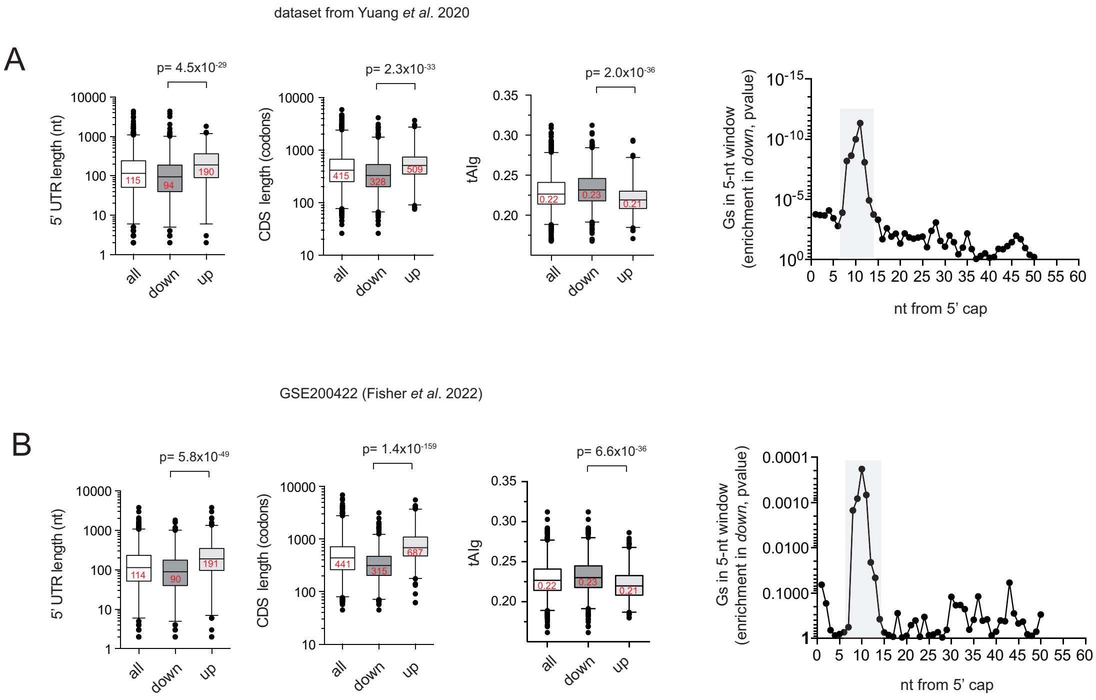

Figure S6. Comparative analysis of 5' UTR length, CDS length (in codons) and tAIg in all, *down* and *up* groups using the indicated datasets of mRNA abundance. Plot boxes represent the median and the 1-99 percentile whiskers; p-values after Mann-Whitney U test are indicated. The enrichment in Gs along the first 50 nt of the 5' UTR in the *down* group of mRNA for every analysis is also shown. Data represent the p-value after a *t*-test comparison between *down* and *up* groups.

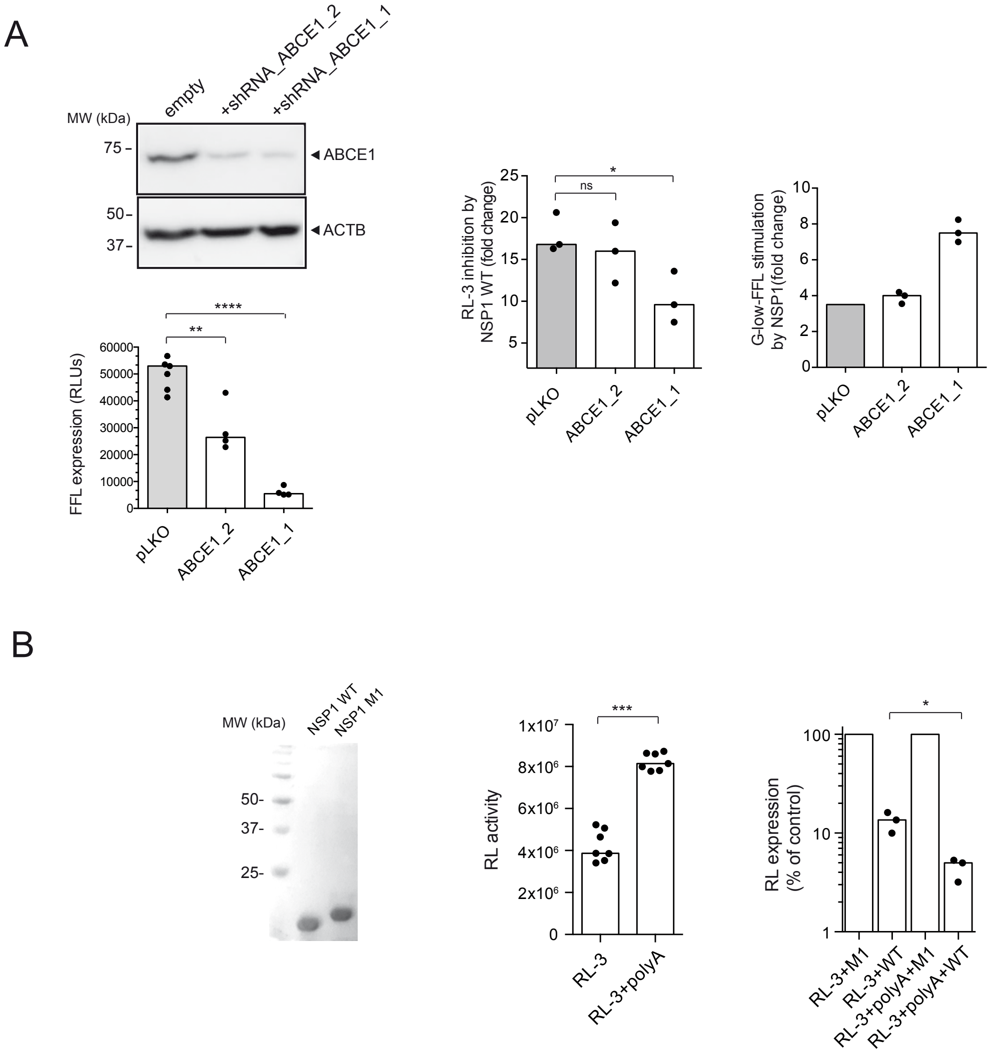

Figure S7. (A) Effect of ABCE1 knockdown on reporters´ sensitivity to NSP1. Western blot analysis of ABCE1 expression in HEK293T cells transduced with lentivirus expressing no shRNA (empty) or the indicated shRNA targeting ABCE1 (left upper panel). The effect of ABCE1 knockdown on expression of FFL is shown (left bottom panel). In middle and right panels, cells were cotransfected with RL-3, Glow-FFL and NSP1 WT or M1, and 24h later RL (middle) and FFL (right) activities were measured. Histograms represent the fold change inhibition or stimulation by NSP1 WT compared to control (M1). (B) Coomassie blue staining of recombinant NSP1s (left panel). In the middle panel, 25 ng of RL-3 or RL-3+polyA mRNAs were translated in RRL supplemented with HEK293T S10 extract and the corresponding RL activities (n=7) were measured 60 min later (middle panel). Right panel shows the effect of NSP1 M1 or WT (0.5 µg) on translation of mRNA with or without polyA tail (n=3). Data are expressed as % of control (+NSP1 M1).

Table S1. Sequence and features of CDSs

reporter	coding sequence	G+C(%)	tAIg	CAI	CSCg
EGFP(parental)	ATGGTGAGCAAGGGCGAGGAGCTGTTCACCGGGGTGGTGCCCATCCTGGTCGAGCTGGACGGCGACGTAAAC<br>GGCCACAAGTTCAGCGTGTCCGGCGAGGGCGAGGGCGATGCCACCTACGGCAAGCTGACCCTGAAGTTCATC<br>TGCACCACCGGCAAGCTGCCCGTGCCCTGGCCCACCCTCGTGACCACCCTGACCTACGGCGTGCAGTGCTTC<br>AGCCGCTACCCCGACCACATGAAGCAGCACGACTTCTTCAAGTCCGCCATGCCCGAAGGCTACGTCCAGGAG<br>CGCACCATCTTCTTCAAGGACGACGGCAACTACAAGACCCGCGCCGAGGTGAAGTTCGAGGGCGACACCCTG<br>GTGAACCGCATCGAGCTGAAGGGCATCGACTTCAAGGAGGACGGCAACATCCTGGGGCACAAGCTGGAGTAC<br>AACTACAACAGCCACAACGTCTATATCATGGCCGACAAGCAGAAGAACGGCATCAAGGTGAACTTCAAGATC<br>CGCCACAACATCGAGGACGGCAGCGTGCAGCTCGCCGACCACTACCAGCAGAACACCCCCATCGGCGACGGC<br>CCCGTGCTGCTGCCCGACAACCACTACCTGAGCACCCAGTCCGCCCTGAGCAAAGACCCCAACGAGAAGCGC<br>GATCACATGGTCCTGCTGGAGTTCGTGACCGCCGCCGGGATCACTCTCGGCATGGACGAGCTGTACAAGTAA	61.53	0.29	0.96	0.20
EGFP-1	ATGGTGTCAAAAGGAGAAGAGCTGTTCACGGGAGTTGTACCAATATTAGTGGAATTAGACGGCGATGTTAAC<br>GGACATAAATTCTCAGTTTCCGGGGAAGGAGAAGGGGATGCCACATACGGTAAATTGACACTTAAATTTATT<br>TGTACAACAGGAAAATTGCCTGTTCCTTGGCCTACATTGGTGACAACATTGACATACGGAGTTCAATGTTTT<br>TCTAGATACCCTGATCACATGAAACAACACGATTTCTTCAAATCGGCCATGCCTGAAGGATACGTCCAGGAA<br>CGCACCATTTTCTTCAAGGATGATGGTAACTACAAAACAAGAGCCGAAGTGAAATTTGAGGGGGATACTTTA<br>GTTAATAGAATCGAATTGAAAGGAATAGACTTCAAAGAGGATGGCAACATTTTGGGACATAAGCTTGAATAC<br>AACTACAATTCCCATAACGTGTATATCATGGCGGATAAACAAAAGAACGGGATTAAGGTAAATTTTAAGATT<br>AGACACAACATAGAAGATGGATCCGTGCAACTGGCCGATCATTACCAACAAAACACACCTATTGGAGATGGA<br>CCTGTTTTGTTACCTGATAACCATTACTTGTCTACACAATCTGCGTTGTCTAAGGATCCTAACGAAAAAAGA<br>GACCATATGGTTCTGTTAGAATTTGTGACAGCAGCTGGCATTACACTAGGTATGGATGAGCTCTACAAATAA	39.72	0.22	0.76	-0.10
EGFP-2	ATGGTTAGTAAAGGTGAGGAACTCTTTACCGGGGTTGTTCCTATTTTGGTTGAATTGGATGGAGACGTCAAC<br>GGACATAAATTTTCTGTCTCTGGAGAAGGAGAAGGAGATGCCACATACGGAAAATTGACATTGAAATTTATT<br>TGTACCACTGGAAAATTGCCTGTTCCATGGCCAACCTTGGTTACAACATTAACATACGGAGTTCAATGTTTT<br>TCTAGATACCCAGATCATATGAAACAACATGATTTTTTTAAAAGTGCGATGCCTGAAGGGTACGTGCAGGAA<br>AGAACAATTTTTTTTAAAGATGACGGAAACTACAAAACAAGAGCCGAAGTTAAATTTGAAGGAGATACATTG<br>GTTAATAGAATTGAATTGAAAGGAATTGATTTTAAAGAAGATGGAAACATTTTAGGACACAAATTGGAATAC<br>AACTACAACTCTCACAATGTTTACATTATGGCAGACAAACAAAAAAACGGAATTAAAGTTAACTTTAAAATT<br>AGACATAACATTGAAGATGGATCCGTGCAATTGGCCGATCATTACCAACAAAATACACCTATTGGAGATGGA<br>CCTGTTTTGTTGCCTGATAACCATTACTTGTCCACACAATCTGCCTTGTCTAAAGACCCGAACGAAAAAAGA<br>GATCATATGGTTTTACTTGAATTTGTTACAGCGGCGGGAATTACTTTGGGCATGGACGAATTATATAAATAA	35.83	0.21	0.76	-0.16
FFL	ATGGAAGACGCCAAAAACATAAAGAAAGGCCCGGCGCCATTCTATCCTCTAGAGGATGGAACCGCTGGAGAG<br>CAACTGCATAAGGCTATGAAGAGATACGCCCTGGTTCCTGGAACAATTGCTTTTACAGATGCACATATCGAG<br>GTGAACATCACGTACGCGGAATACTTCGAAATGTCCGTTCGGTTGGCAGAAGCTATGAAACGATATGGGCTG<br>AATACAAATCACAGAATCGTCGTATGCAGTGAAAACTCTCTTCAATTCTTTATGCCGGTGTTGGGCGCGTTA	44.76	0.22	0.71	-0.06

	TTTATCGGAGTTGCAGTTGCGCCCGCGAACGACATTTATAATGAACGTGAATTGCTCAACAGTATGAACATT<br>TCGCAGCCTACCGTAGTGTTTGTTTCCAAAAAGGGGTTGCAAAAAATTTTGAACGTGCAAAAAAAATTACCA<br>ATAATCCAGAAAATTATTATCATGGATTCTAAAACGGATTACCAGGGATTTCAGTCGATGTACACGTTCGTC<br>ACATCTCATCTACCTCCCGGTTTTAATGAATACGATTTTGTACCAGAGTCCTTTGATCGTGACAAAACAATT<br>GCACTGATAATGAATTCCTCTGGATCTACTGGGTTACCTAAGGGTGTGGCCCTTCCGCATAGAACTGCCTGC<br>GTCAGATTCTCGCATGCCAGAGATCCTATTTTTGGCAATCAAATCATTCCGGATACTGCGATTTTAAGTGTT<br>GTTCCATTCCATCACGGTTTTGGAATGTTTACTACACTCGGATATTTGATATGTGGATTTCGAGTCGTCTTA<br>ATGTATAGATTTGAAGAAGAGCTGTTTTTACGATCCCTTCAGGATTACAAAATTCAAAGTGCGTTGCTAGTA<br>CCAACCCTATTTTCATTCTTCGCCAAAAGCACTCTGATTGACAAATACGATTTATCTAATTTACACGAAATT<br>GCTTCTGGGGGCGCACCTCTTTCGAAAGAAGTCGGGGAAGCGGTTGCAAAACGCTTCCATCTTCCAGGGATA<br>CGACAAGGATATGGGCTCACTGAGACTACATCAGCTATTCTGATTACACCCGAGGGGGATGATAAACCGGGC<br>GCGGTCGGTAAAGTTGTTCCATTTTTTGAAGCGAAGGTTGTGGATCTGGATACCGGGAAAACGCTGGGCGTT<br>AATCAGAGAGGCGAATTATGTGTCAGAGGACCTATGATTATGTCCGGTTATGTAAACAATCCGGAAGCGACC<br>AACGCCTTGATTGACAAGGATGGATGGCTACATTCTGGAGACATAGCTTACTGGGACGAAGACGAACACTTC<br>TTCATAGTTGACCGCTTGAAGTCTTTAATTAAATACAAAGGATATCAGGTGGCCCCCGCTGAATTGGAATCG<br>ATATTGTTACAACACCCCAACATCTTCGACGCGGGCGTGGCAGGTCTTCCCGACGATGACGCCGGTGAACTT<br>CCCGCCGCCGTTGTTGTTTTGGAGCACGGAAAGACGATGACGGAAAAAGAGATCGTGGATTACGTCGCCAGT<br>CAAGTAACAACCGCGAAAAAGTTGCGCGGAGGAGTTGTGTTTGTGGACGAAGTACCGAAAGGTCTTACCGGA<br>AAACTCGACGCAAGAAAAATCAGAGAGATCCTCATAAAGGCCAAGAAGGGCGGAAAGTCCAAATTGTAA				
RL (parental)	ATGGAACAAAAACTCATCTCAGAAGAGGATCTGTCGAGCTCCACTTCGAAAGTTTATGATCCAGAACAAAGG<br>AAACGGATGATAACTGGTCCGCAGTGGTGGGCCAGATGTAAACAAATGAATGTTCTTGATTCATTTATTAAT<br>TATTATGATTCAGAAAAACATGCAGAAAATGCTGTTATTTTTTTACATGGTAACGCGGCCTCTTCTTATTTA<br>TGGCGACATGTTGTGCCACATATTGAGCCAGTAGCGCGGTGTATTATACCAGACCTTATTGGTATGGGCAAA<br>TCAGGCAAATCTGGTAATGGTTCTTATAGGTTACTTGATCATTACAAATATCTTACTGCATGGTTTGAACTT<br>CTTAATTTACCAAAGAAGATCATTTTTGTCGGCCATGATTGGGGTGCTTGTTTGGCATTTCATTATAGCTAT<br>GAGCATCAAGATAAGATCAAAGCAATAGTTCACGCTGAAAGTGTAGTAGATGTGATTGAATCATGGGATGAA<br>TGGCCTGATATTGAAGAAGATATTGCGTTGATCAAATCTGAAGAAGGAGAAAAAATGGTTTTGGAGAATAAC<br>TTCTTCGTGGAAACCATGTTGCCATCAAAAATCATGAGAAAGTTAGAACCAGAAGAATTTGCAGCATATCTT<br>GAACCATTCAAAGAGAAAGGTGAAGTTCGTCGTCCAACATTATCATGGCCTCGTGAAATCCCGTTAGTAAAA<br>GGTGGTAAACCTGACGTTGTACAAATTGTTAGGAATTATAATGCTTATCTACGTGCAAGTGATGATTTACCA<br>AAAATGTTTATTGAATCGGACCCAGGATTCTTTTCCAATGCTATTGTTGAAGGTGCCAAGAAGTTTCCTAAT<br>ACTGAATTTGTCAAAGTAAAAGGTCTTCATTTTTCGCAAGAAGATGCACCTGATGAAATGGGAAAATATATC<br>AAATCGTTCGTTGAGCGAGTTCTCAAAAATGAACAATAA	36.92	0.20	0.68	-0.15
RL-1	ATGGAACAAAAACTTATTTCAGAGGAGGACCTCTCTTCAAGCACCAGCAAGGTGTACGACCCTGAGCAGCGT<br>AAGAGAATGATCACCGGGCCACAATGGTGGGCCAGATGCAAGCAGATGAATGTGCTGGACAGCTTCATCAAC<br>TATTACGACAGCGAGAAGCACGCCGAGAACGCCGTGATCTTCCTGCACGGCAACGCGGCAAGTAGCTACCTG<br>TGGAGACACGTGGTACCTCACATTGAACCTGTTGCTAGGTGCATAATTCCCGATCTGATTGGAATGGGAAAG	52.72	0.25	0.84	0.07

	TCCGGAAAGTCCGGCAATGGAAGCTACAGGCTGCTGGATCATTACAAGTACCTGACCGCTTGGTTCGAGCTG<br>TTGAACCTGCCAAAAAAAATTATTTTTGTTGGACACGATTGGGGCGCCTGCCTTGCCTTCCATTACTCCTAT<br>GAGCACCAAGACAAGATTAAAGCCATTGTGCACGCAGAATCCGTCGTCGACGTGATCGAGAGCTGGGACGAG<br>TGGCCCGACATCGAGGAGGATATTGCCCTGATTAAGAGCGAAGAGGGGGAGAAGATGGTTCTCGAGAACAAC<br>TTCTTCGTGGAAACTATGTTGCCGTCGAAGATTATGAGAAAGCTGGAGCCCGAGGAGTTCGCTGCATACCTC<br>GAGCCCTTCAAGGAGAAGGGTGAGGTCAGGCGGCCAACCCTGTCTTGGCCCCGCGAGATTCCCTTGGTCAAG<br>GGGGGCAAGCCCGACGTGGTCCAGATCGTTCGGAACTACAACGCCTACCTGCGGGCTAGTGACGACCTGCCA<br>AAGATGTTCATTGAGAGCGACCCAGGGTTCTTCTCGAACGCCATCGTGGAGGGGGCGAAGAAATTTCCCAAT<br>ACCGAATTCGTGAAGGTGAAGGGGCTTCACTTCAGCCAGGAGGACGCCCCGGACGAAATGGGCAAGTACATC<br>AAGTCCTTCGTGGAACGCGTACTAAAAAATGAACAATAA				
RL-2	ATGGAGCAGAAACTAATAAGTGAGGAGGATCTCTCTTCTTCTACATCTAAAGTTTACGATCCTGAACAGCGA<br>AAACGAATGATTACAGGGCCTCAGTGGTGGGCGCGCTGTAAACAAATGAATGTTTTGGATTCTTTTATTAAC<br>TACTACGATTCTGAAAAACATGCGGAAAATGCTGTTATCTTTTTGCATGGAAACGCCGCCTCCTCTTACTTA<br>TGGCGTCACGTAGTCCCTCATATTGAACCTGTAGCCCGGTGCATCATTCCTGATTTAATAGGAATGGGCAAA<br>TCTGGAAAATCTGGGAACGGATCTTACAGACTGCTGGATCATTACAAATACTTGACAGCCTGGTTTGAACTG<br>CTAAATTTACCTAAAAAAATTATCTTTGTGGGCCATGATTGGGGGGCCTGCCTTGCCTTTCATTACTCGTAT<br>GAACATCAGGACAAAATCAAAGCAATAGTGCATGCCGAATCAGTGGTTGATGTCATTGAATCCTGGGATGAA<br>TGGCCTGACATTGAAGAAGATATTGCCTTGATCAAAAGTGAGGAAGGAGAAAAAATGGTATTGGAAAACAAC<br>TTCTTTGTAGAGACAATGCTGCCGTCTAAAATTATGCGTAAATTAGAACCTGAAGAATTTGCTGCGTACTTG<br>GAACCTTTTAAAGAAAAAGGGGAAGTTCGTAGACCTACTCTGAGCTGGCCTAGAGAAATTCCACTAGTTAAA<br>GGAGGAAAACCTGATGTCGTGCAGATTGTAAGAAACTACAACGCCTACCTGCGCGCCTCTGATGATTTGCCT<br>AAAATGTTTATTGAATCTGATCCTGGGTTCTTCTCAAATGCCATTGTTGAAGGAGCTAAGAAATTCCCTAAC<br>ACGGAATTTGTGAAAGTGAAAGGACTTCATTTTTCTCAAGAAGATGCTCCAGACGAAATGGGAAAATACATT<br>AAATCCTTTGTCGAGCGCGTATTGAAAAACGAGCAATAA	40.72	0.21	0.75	-0.10
RL-3	ATGGAGCAGAAGCTGATCAGCGAGGAGGACCTGAGCAGCAGCACCAGCAAGGTGTACGACCCCGAGCAGAGA<br>AAGAGAATGATCACCGGCCCCCAGTGGTGGGCCAGATGCAAGCAGATGAACGTGCTGGACAGCTTCATCAAC<br>TACTACGACAGCGAGAAGCACGCCGAGAACGCCGTGATCTTCCTGCACGGCAACGCCGCCAGCAGCTACCTG<br>TGGAGACACGTGGTGCCCCACATCGAGCCCGTGGCCAGATGCATCATCCCCGACCTGATCGGCATGGGCAAG<br>AGCGGCAAGAGCGGCAACGGCAGCTACAGACTGCTGGACCACTACAAGTACCTGACCGCCTGGTTCGAGCTG<br>CTGAACCTGCCCAAGAAGATCATCTTCGTGGGCCACGACTGGGGCGCCTGCCTGGCCTTCCACTACAGCTAC<br>GAGCACCAGGACAAGATCAAGGCCATCGTGCACGCCGAGAGCGTGGTGGACGTGATCGAGAGCTGGGACGAG<br>TGGCCCGACATCGAGGAGGACATCGCCCTGATCAAGAGCGAGGAGGGCGAGAAGATGGTGCTGGAGAACAAC<br>TTCTTCGTGGAGACCATGCTGCCCAGCAAGATCATGAGAAAGCTGGAGCCCGAGGAGTTCGCCGCCTACCTG<br>GAGCCCTTCAAGGAGAAGGGCGAGGTGAGAAGACCCACCCTGAGCTGGCCCAGAGAGATCCCCCTGGTGAAG<br>GGCGGCAAGCCCGACGTGGTGCAGATCGTGAGAAACTACAACGCCTACCTGAGAGCCAGCGACGACCTGCCC<br>AAGATGTTCATCGAGAGCGACCCCGGCTTCTTCAGCAACGCCATCGTGGAGGGCGCCAAGAAGTTCCCCAAC<br>ACCGAGTTCGTGAAGGTGAAGGGCCTGCACTTCAGCCAGGAGGACGCCCCCGACGAGATGGGCAAGTACATC<br>AAGAGCTTCGTGGAGAGAGTGCTGAAGAACGAGCAGTAA	60.36	0.29	1.00	0.19

nanoLuc	ATGGTCTTCACACTCGAAGATTTCGTTGGGGACTGGCGACAGACAGCCGGCTACAACCTGGACCAAGTCCTT<br>GAACAGGGAGGTGTGTCCAGTTTGTTTCAGAATCTCGGGGTGTCCGTAACTCCGATCCAAAGGATTGTCCTG<br>AGCGGTGAAAATGGGCTGAAGATCGACATCCATGTCATCATCCCGTATGAAGGTCTGAGCGGCGACCAAATG<br>GGCCAGATCGAAAAAATTTTTAAGGTGGTGTACCCTGTGGATGATCATCACTTTAAGGTGATCCTGCACTAT<br>GGCACACTGGTAATCGACGGGGTTACGCCGAACATGATCGACTATTTCGGACGGCCGTATGAAGGCATCGCC<br>GTGTTCGACGGCAAAAAGATCACTGTAACAGGGACCCTGTGGAACGGCAACAAAATTATCGACGAGCGCCTG<br>ATCAACCCCGACGGCTCCCTGCTGTTCCGAGTAACCATCAACGGAGTGACCGGCTGGCGGCTGTGCGAACGC<br>ATTCTGGCGTAA	52.71	0.26	0.80	0.06
dsRED2	ATGGCCTCCTCCGAGAACGTCATCACCGAGTTCATGCGCTTCAAGGTGCGCATGGAGGGCACCGTGAACGGC<br>CACGAGTTCGAGATCGAGGGCGAGGGCGAGGGCCGCCCCTACGAGGGCCACAACACCGTGAAGCTGAAGGTG<br>ACCAAGGGCGGCCCCCTGCCCTTCGCCTGGGACATCCTGTCCCCCCAGTTCCAGTACGGCTCCAAGGTGTAC<br>GTGAAGCACCCCGCCGACATCCCCGACTACAAGAAGCTGTCCTTCCCCGAGGGCTTCAAGTGGGAGCGCGTG<br>ATGAACTTCGAGGACGGCGGCGTGGCGACCGTGACCCAGGACTCCTCCCTGCAGGACGGCTGCTTCATCTAC<br>AAGGTGAAGTTCATCGGCGTGAACTTCCCCTCCGACGGCCCCGTGATGCAGAAGAAGACCATGGGCTGGGAG<br>GCCTCCACCGAGCGCCTGTACCCCCGCGACGGCGTGCTGAAGGGCGAGACCCACAAGGCCCTGAAGCTGAAG<br>GACGGCGGCCACTACCTGGTGGAGTTCAAGTCCATCTACATGGCCAAGAAGCCCGTGCAGCTGCCCGGCTAC<br>TACTACGTGGACGCCAAGCTGGACATCACCTCCCACAACGAGGACTACACCATCGTGGAGCAGTACGAGCGC<br>ACCGAGGGCCGCCACCACCTGTTCCTGTAG	63.72	0.30	0.98	0.22
ACTB*	ATGGTGGGCATGGGTCAGAAGGATTCCTATGTGGGCGACGAGGCCCAGAGCAAGAGAGGCATCCTCACCCTG<br>AAGTACCCCATCGAGCACGGCATCGTCACCAACTGGGACGACATGGAGAAAATCTGGCACCACACCTTCTAC<br>AATGAGCTGCGTGTGGCTCCCGAGGAGCACCCCGTGCTGCTGACCGAGGCCCCCCTGAACCCCAAGGCCAAC<br>CGCGAGAAGATGACCCAGATCATGTTTGAGACCTTCAACACCCCAGCCATGTACGTTGCTATCCAGGCTGTG<br>CTATCCCTGTACGCCTCTGGCCGTACCACTGGCATCGTGATGGACTCCGGTGACGGGGTCACCCACACTGTG<br>CCCATCTACGAGGGGTATGCCCTCCCCCATGCCATCCTGCGTCTGGACCTGGCTGGCCGGGACCTGACTGAC<br>TACCTCATGAAGATCCTCACCGAGCGCGGCTACAGCTTCACCACCACGGCCGAGCGGGAAATCGTGCGTGAC<br>ATTAAGGAGAAGCTGTGCTACGTCGCCCTGGACTTCGAGCAAGAGATGGCCACGGCTGCTTCCAGCTCCTCC<br>CTGGAGAAGAGCTACGAGCTGCCTGACGGCCAGGTCATCACCATTGGCAATGAGCGGTTCCGCTGCCCTGAG<br>GCACTCTTCCAGCCTTCCTTCCTGGGCATGGAGTCCTGTGGCATCCACGAAACTACCTTCAACTCCATCATG<br>AAGTGTGACGTGGACATCCGCAAAGACCTGTACGCCAACACAGTGCTGTCTGGCGGCACCACCATGTACCCT<br>GGCATTGCCGACAGGATGCAGAAGGAGATCACTGCCCTGGCACCCAGCACAATGAAGATCAAGATCATTGCT<br>CCTCCTGAGCGCAAGTACTCCGTGTGGATCGGCGGCTCCATCCTGGCCTCGCTGTCCACCTTCCAGCAGATG<br>TGGATCAGCAAGCAGGAGTAG	55.87	0.25	0.88	0.11
N SARS2	ATGTCTGATAATGGACCCCAAAATCAGCGAAATGCACCCCGCATTACGTTTGGTGGACCCTCAGATTCAACT<br>GGCAGTAACCAGAATGGAGAACGCAGTGGGGCGCGATCAAAACAACGTCGGCCCCAAGGTTTACCCAATAAT	47.22	0.20	0.74	-0.08

	ACTGCGTCTTGGTTCACCGCTCTCACTCAACATGGCAAGGAAGACCTTAAATTCCCTCGAGGACAAGGCGTT<br>CCAATTAACACCAATAGCAGTCCAGATGACCAAATTGGCTACTACCGAAGAGCTACCAGACGAATTCGTGGT<br>GGTGACGGTAAAATGAAAGATCTCAGTCCAAGATGGTATTTCTACTACCTAGGAACTGGGCCAGAAGCTGGA<br>CTTCCCTATGGTGCTAACAAAGACGGCATCATATGGGTTGCAACTGAGGGAGCCTTGAATACACCAAAAGAT<br>CACATTGGCACCCGCAATCCTGCTAACAATGCTGCAATCGTGCTACAACTTCCTCAAGGAACAACATTGCCA<br>AAAGGCTTCTACGCAGAAGGGAGCAGAGGCGGCAGTCAAGCCTCTTCTCGTTCCTCATCACGTAGTCGCAAC<br>AGTTCAAGAAATTCAACTCCAGGCAGCAGTAGGGGAACTTCTCCTGCTAGAATGGCTGGCAATGGCGGTGAT<br>GCTGCTCTTGCTTTGCTGCTGCTTGACAGATTGAACCAGCTTGAGAGCAAAATGTCTGGTAAAGGCCAACAA<br>CAACAAGGCCAAACTGTCACTAAGAAATCTGCTGCTGAGGCTTCTAAGAAGCCTCGGCAAAAACGTACTGCC<br>ACTAAAGCATACAATGTAACACAAGCTTTCGGCAGACGTGGTCCAGAACAAACCCAAGGAAATTTTGGGGAC<br>CAGGAACTAATCAGACAAGGAACTGATTACAAACATTGGCCGCAAATTGCACAATTTGCCCCCAGCGCTTCA<br>GCGTTCTTCGGAATGTCGCGCATTGGCATGGAAGTCACACCTTCGGGAACGTGGTTGACCTACACAGGTGCC<br>ATCAAATTGGATGACAAAGATCCAAATTTCAAAGATCAAGTCATTTTGCTGAATAAGCATATTGACGCATAC<br>AAAACATTCCCACCAACAGAGCCTAAAAAGGACAAAAAGAAGAAGGCTGATGAAACTCAAGCCTTACCGCAG<br>AGACAGAAGAAACAGCAAACTGTGACTCTTCTTCCTGCTGCAGATTTGGATGATTTCTCCAAACAATTGCAA<br>CAATCCATGAGCAGTGCTGACTCAACTCAGGCCTAAT				
N1 SARS2	ATGAGCGACAATGGCCCCCAGAACCAGAGAAACGCTCCTCGCATCACCTTCGGCGGCCCATCTGATAGCACC<br>GGGAGCAATCAGAATGGCGAGAGGAGCGGGGCCAGATCAAAACAGAGGAGACCCCAGGGGCTGCCAAACAAT<br>ACCGCCAGCTGGTTTACTGCCCTGACTCAGCACGGCAAGGAGGATCTGAAGTTCCCTAGGGGTCAGGGCGTG<br>CCTATCAATACAAACTCTAGCCCCGATGACCAGATCGGATACTACCGCCGCGCTACACGGAGAATCAGGGGC<br>GGCGATGGAAAAATGAAAGACCTGTCTCCCCGGTGGTACTTCTACTATCTGGGCACCGGCCCTGAAGCTGGA<br>CTTCCCTATGGTGCCAACAAGGACGGAATCATTTGGGTGGCCACCGAAGGCGCCCTGAATACACCAAAGGAC<br>CACATCGGCACCAGGAATCCTGCTAACAATGCTGCAATCGTGCTGCAGCTGCCCCAGGGAACTACCCTGCCT<br>AAGGGTTTCTACGCTGAAGGCTCCCGCGGGGGCTCCCAGGCCTCCAGCAGGTCTTCCAGCAGATCCCGCAAT<br>TCCTCCCGCAATAGCACCCCCGGCTCCTCTCGGGGCACCAGCCCAGCCAGGATGGCTGGAAATGGCGGCGAC<br>GCCGCTCTTGCCCTGCTGCTGCTGGACAGGCTGAATCAGCTGGAGTCTAAGATGAGCGGGAAGGGCCAGCAG<br>CAGCAGGGCCAGACCGTGACCAAGAAGTCCGCAGCCGAGGCCAGCAAGAAGCCCAGGCAGAAAAGAACAGCC<br>ACAAAAGCCTACAACGTCACTCAGGCCTTTGGCAGGAGGGGACCCGAACAGACTCAGGGCAACTTTGGCGAC<br>CAGGAGCTGATCCGCCAGGGAACCGACTACAAGCACTGGCCTCAGATCGCCCAGTTCGCCCCCTCTGCCAGC<br>GCTTTTTTTGGCATGAGCAGGATCGGAATGGAGGTGACTCCAAGCGGCACCTGGCTGACTTACACCGGGGCT<br>ATTAAGCTGGACGACAAAGATCCCAACTTCAAGGATCAGGTGATCCTCCTGAACAAGCACATCGACGCCTAC<br>AAGACCTTCCCCCCTACCGAGCCTAAGAAGGATAAGAAGAAAAAGGCCGACGAGACCCAGGCCCTCCCTCAG<br>AGACAGAAAAAGCAGCAGACCGTGACCCTGCTGCCTGCCGCCGATCTGGACGATTTCTCTAAACAGCTGCAG<br>CAGAGCATGAGTTCCGCCGACAGTACCCAGGCCTGA	59.00	0.25	0.92	0.08

Table S2. Sequence of 5' UTRs

5' UTR	sequence	reporter
94	AGATCCGCTAGCGCTACCGGACTCAGATCTCGAGCTCAAGCTTCGAATTCTGCAGTCGACGGTACCGCGGGCCC<br>GGGATCCACCGGTCGCCACC**ATG**	EGFP,dsRed2,<br>nanoLuc,N<br>SARS-CoV-2
82	AGATCCGCTAGCGCTACCGGACTCAGATCTCGAGCTCAAGCTTCGAATTCTGCAGTCGACGGTACCGCGGGCCC<br>GGGATCCA**ATG**	FFL, RL
223	AGCGCTACCGGACTCAGAATCTCGAGCTCAAGCTTCGAATTCTGCAGTCGACGGTACCGCGGGCCCCTTGGCCT<br>GGCTTCGCGCTACGCCGGCGCGCGCGCGGCCGCGAATTGTAGGTGGGCGTGGCCTCCAAGGGCGTGGCGGCATT<br>CGTGGTCTCCATCGCCTGCCATAAAACACTTGTGTTGGTAGGAAATCCATAGAGCGCCCCTATAGTGGGGATCC<br>A**ATG**	RL
424	AGATCCGCTAGCGCTACCGGACTCAGATCTCGAGCTCTCTGGCTAACTAGGGAACCCACTGCTTAAGCCTCAAT<br>AAAGCTTGCCTTGAGTGCTTCAAGTAGTGTGTGCCCGTCTGTTGTGTGACTCTGGTAACTAGAGATCCCTCAGA<br>CCCTTTTAGTCAGTGTGGAAAATCTCTAGCAGTGGCGCCCGAACAGGGACTTGAAAGCGAAAGGGAAACCAGAG<br>GAGCTCAAGCTTCGAATTCTGCAGTCGACGGTACCGCGGGCCCCTTGGCCTGGCTTCGCTCTACGCCGGCGCGC<br>GCGCGGCGCGAATTGTAGGTGGGCGTGGCCTCCAAGGGCGTGGCGGCATTCGTGGTCTCCATCGCCTGCCATAA<br>AACACTTGTGTTGGTAGGAAATCCATTCTAGAGCGCCCCTATAGTGGGGATCCA**ATG**	RL
626	AGATCTCGAGCTCAAGCTTCGAATTCTGCAGTCGACGGTACCGCGGGCCCAGGTAGACAATATTACACCTGTCC<br>TACTGGCATTGAGAACTTTTGCCCAGAGCAAAAGAGCATTCCAAGCCATCAGAGGGGAAATAAAGCATCTCTAC<br>GGTGGTCCTAAATAGTCAGCATAGTACATTTCATCTGACTAATACTACAACACCACCACCTCTAGCGCTACCGG<br>ACTCAGATCTCGAGCTCTCTGGCTAACTAGGGAACCCACTGCTTAAGCCTCAATAAAGCTTGCCTTGAGTGCTT<br>CAAGTAGTGTGTGCCCGTCTGTTGTGTGACTCTGGTAACTAGAGATCCCTCAGACCCTTTTAGTCAGTGTGGAA<br>AATCTCTAGCAGTGGCGCCCGAACAGGGACTTGAAAGCGAAAGGGAAACCAGAGGAGCTCAAGCTTCGAATTCT<br>GCAGTCGACGGTACCGCGGGCCCCTTGGCCTGGCTTCGCTCTACGCCGGCGCGCGCGCGGCGCGAATTGTAGGT<br>GGGCGTGGCCTCCAAGGGCGTGGCGGCATTCGTGGTCTCCATCGCCTGCCATAAAACACTTGTGTTGGTAGGAA<br>ATCCATTCTAGAGCGCCCCTATAGTGGGGATCCA**ATG**	RL
SL20	AGATCCGCTAGCGCTACCGGACTCAGATCTCGAGCTCAAGCTTCGAATTCTGCAGTCGACGGTACCGCGGGCCC<br>CGACCCGGGCCCGCGGTACGCCGATAGGCGTACGGGATCCA**ATG**	FFL,RL
SL50	AGATCCGCTAGCGCTACCGGACTCAGATCTCGAGCTCAAGCTTCGAATTCTGCAGTCGACCCGGGCCCGCGGAG<br>TACTCCGCGGGCCCGCGGAGTACCGCGGGCCCGGGATCCA**ATG**	FFL,RL
TISU	AGATCAAG**ATG**	EGFP,RL
Gless	AGCCACTATCTCACACCTTTCCTCACTCTTTCCTCACACTTCTTTCTACACTCTTCACAAAAAATAATTTCTCA	RL,RL3

	CTTCCTATTCTTCTCCCCCATCCCTCATTCCTCAATCATTCCTTCCCCATTCACTTCAATCATTCCAA**ATG**	
G-SL1	AGAAAAGATAGTGGAAACCACTATCTCACACCTTTCCTCACTCTTTCCTCACACTTCTTTCTACACTCTTCACA AAAAATAATTTCTCACTTCCTATTCTTCTCCCCCATCCCTCATTCCTCAATCATTCCTTCCCATCCAA**ATG**	RL
G-SL3	AGCCACTATCTCACACCTTTCCTCACTCTTTCCTCACACTTCTTTCTACACTCTTCACAAAAAATAATTTCTCA CTTCCTATTCTTCTCCCCCATCCCTCATTCCTCAATCATTCCGAGATAGTGGAAACCACTATCTCCAA**ATG**	RL
G-M1	AGCCACGAGCTCGGAGCTTGCCGCACTCGGTCCTCACACTTCTTTCTACACTCTTCACAAAAAATAATTTCTCA CTTCCTATTCTTCTCCCCCATCCCTCATTCCTCAATCATTCCTTCCCCATTCACTTCAATCATTCCAA**ATG**	RL
G-M2	AGCCACTATCTCACACCTTTCCTCACTCTTTCCTCACACTTCTTTCTACACTCTTCACAAAAAATAATTTCTCA CTTCCTATTCTTCTCCCCCATCCCTCATTCCTCAATCATGGCGACGCCATTGACTTTGATCATTCCAA**ATG**	RL
G-M3	AGCCACGAGCTCGGAGCTTGCCGCACTCGGTCCTCACACTTCTTTCTACACTCTTCACAAAAAATAATTTCTCA CTTCCTATTCTTCTCCCCCATCCCTCATTCCTCAATCATGGCGACGCCATTGACTTTGATCATTCCAA**ATG**	RL
G-M4	AGCCACGAGAGAGAGAGATTCCTCACTCTTTCCTCACACTTCTTTCTACACTCTTCACAAAAAATAATTTCTCA CTTCCTATTCTTCTCCCCCATCCCTCATTCCTCAATCATTCCTTCCCCATTCACTTCAATCATTCCAA**ATG**	RL
Slot	AGCACAACAACAACAAACCCTCGAACAACAACAACAACAACAACAACACC**ATG**	RL
Gless_8	AGCCACAA**ATG**	RL
Gless_12	AGCCACTTCCAA**ATG**	RL
Gless_15	AGCCACTATTTCCAA**ATG**	RL
Gless_22	AGCCACTATCTCATCATTCCAA**ATG**	RL
Gless_30	AGCCACTATCTCACACCTCAATCATTCCAA**ATG**	RL

Gless_67	AGCCACTATCTCACACCTTTCCTCACTCTTTCCTCCATTCCTTCCCCATTCACTTCAATCATTCCAA**ATG**	RL
5'L-N	ATTAAAGGTTTATACCTTCCCAGGTAACAAACCAACCAACTTTCGATCTCTTGTAGATCTGTTCTCTAAACGAA<br>CAAACT**ATG**	RL, N SARS2
5'L(Gs)-N	ATTAAAGGTGGAGACGAGCCGAGGTAACAAACCAACCAACTTTCGATCTCTTGTAGATCTGTTCTCTAAACGAA<br>CAAACT**ATG**	RL, N SARS2
G-uATG_8	AGCCACTATGCACACCTTTCCTCACTCTTTCCTCACACTTCTTTCTACACTCTTCACAAAAAATAATTTCTCAC<br>TTCCTATTCTTCTCCCCCATCCCTCATTCCTCAATCATTCCTTCCCCATTCACTTCAATCATTCCAA**ATG**	RL
G-uATG_13	AGCCACTATATCATGCCCTTTCCTCACTCTTTCCTCACACTTCTTTCTACACTCTTCACAAAAAATAATTTCTC<br>ACTTCCTATTCTTCTCCCCCATCCCTCATTCCTCAATCATTCCTTCCCCATTCACTTCAATCATTCCAA**ATG**	RL
G-uATG_18	AGCCACTATCTCACACCATGCCTCACTCTTTCCTCACACTTCTTTCTACACTCTTCACAAAAAATAATTTCTCA<br>CTTCCTATTCTTCTCCCCCATCCCTCATTCCTCAATCATTCCTTCCCCATTCACTTCAATCATTCCAA**ATG**	RL
G-uATG_28	AGCCACTATCTCACACCTTTCCTCACTATGATCCTCACACTTCTTTCTACACTCTTCACAAAAAATAATTTCTC<br>ACTTCCTATTCTTCTCCCCCATCCCTCATTCCTCAATCATTCCTTCCCCATTCACTTCAATCATTCCAA**ATG**	RL
G-uATG_112	AGCCACTATCTCACACCTTTCCTCACTCTTTCCTCACACTTCTTTCTACACTCTTCACAAAAAATAATTTCTCA<br>CTTCCTATTCTTCTCCCCCATCCCTCATTCCTCAATCATGCCTTCCCCATTCACTTCAATCATTCCAA**ATG**	RL
G-M4-uATG_8	AGCCACGATGAAGAGAGAGAGATTCCTCACTCTTTCCTCACACTTCTTTCTACACTCTTCACAAAAAATAATTTCT<br>CACTTCCTATTCTTCTCCCCCATCCCTCATTCCTCAATCATTCCTTCCCCATTCACTTCAATCATTCCAA**ATG**	RL
G-M4-uATG_28	AGCCACGAGAGAGAGAGATTCCTCACTATGATCCTCACACTTCTTTCTACACTCTTCACAAAAAATAATTTCTC<br>ACTTCCTATTCTTCTCCCCCATCCCTCATTCCTCAATCATTCCTTCCCCATTCACTTCAATCATTCCAA**ATG**	RL
G-low	AGATCCGGCTTATTTCCTCCTTCTCCACTATCTCACACCTTTCCTCACTCTTTCCTCACACTTCTTTCTACACT<br>CTTCACAAAAAATAATTTCTCACTTCCTATTCTTCTCCCCCATCCCTCATTCCTCAATCATTCCTTCCCCATTC<br>ACTTCAATCATTCCAAGGATCCA**ATG**	FFL, RL, RL3,<br>EGFP

Table S3. Sequence of oligonucleotides

oligo name	oligo sequence	purpose
5'-EX-NSP1	CACAATCTCGAGAATTCATGGAGAGCCTGGTGCCTGGCTTCAACG	cloning
3'-NB-NSP1	CACACGGATCCGCGGCCGCTTAGCCACCGTTCAGTTCACGCATCAGC	cloning
5'-N-hACTB	GACGACCCATGGTGGGCATGGGTCAGAAGGATTCC	cloning
3'-X-hACTB	CACCACATCTAGACTAGAAGCATTTGCGGTGGACGATGGA	cloning
5'-161 pEGFPN1	GGGACTTTCCATTGACGTCAATGGGTGG	cloning
3'-TISU-SacI	CTGGTTGCGGAGCTCACCGCCGCCATCTTGATCTGACGGTT	cloning
5'-NanoL	CGACAGGATCCACCATGGTCTTCACACTCGAAGATTTC	cloning
3'-NanoL	GTGTGTGCGGCCGCTACGCCAGAATGCGTTCGCAC	cloning
5'-18S_9	CGGGGAGGTAGTGACG	RT-PCR 18S
3'-18S_4	CGGTATCCAGGCGGCTCGG	RT-PCR 18S
5'-EGFP	GAACCGCATCGAGCTGAA	RT-PCR EGFP
3'-EGFP	TGCTTGTCGGCCATGATATAG	RT-PCR EGFP
5'-RL	GTTAGGAATTATAATGCTTATCTACG	RT-PCR RL
3'-RL	CTTGGCACCTTCAACAATAGC	RT-PCR RL
5'-RL3	GGAGAAGGGCGAGGTGAGAAG	RT-PCR RL3
3'-RL3	CGATGGCGTTGCTGAAGAAG	RT-PCR RL3
5'-NanoL_qPCR	GGAGGTGTGTCCAGTTTGTT	RT-PCR nanoL
3'-NanoL_qPCR	ATGTCGATCTTCAGCCCATTT	RT-PCR nanoL
5'-dsRed	GAACGTCATCACCGAGTTCA	RT-PCR dsRed
3'-dsRed	GGGTGCTTCACGTACACCTT	RT-PCR dsRed
5'-N-SARS2	AAGCTGGACTTCCCTATGGTG	RT-PCR N SARS2
3'-N-SARS2	CGATTGCAGCATTGTTAGCAGG	RT-PCR N SARS2
